# Mesenchymal stem cells have ameliorative effect on the colitis model via Nrf2/HO-1 pathway

**DOI:** 10.1590/acb370704

**Published:** 2022-10-10

**Authors:** Mehmet Fatih Bozkurt, Muhammed Nasir Bhaya, Cengiz Dibekoğlu, Ayberk Akat, Utku Ateş, Oytun Erbaş

**Affiliations:** 1 PhD, assistant professor. Afyon Kocatepe University – Department of Pathology – Faculty of Veterinary Medicine – Afyonkarahisar, Turkey.; 2 PhD, research scholar. Afyon Kocatepe University – Department of Pathology – Faculty of Veterinary Medicine – Afyonkarahisar, Turkey.; 3 MD, PhD. Istanbul Florence Nightingale Hospital – Department of General Surgery – Istanbul, Turkey.; 4 PhD. Stembio Cell and Tissue Technologies Inc. – Istanbul, Turkey.; 5 PhD, professor. Stembio Cell and Tissue Technologies Inc. – Istanbul, Turkey.; 6 PhD, associate professor. Demiroğlu Bilim University – Department of Physiology – Cephalink Institute – Gebze, Turkey.

**Keywords:** Inflammation, Acetic Acid, Mesenchymal Stem Cells, NF-E2-Related Factor 2, Vascular Endothelial Growth Factor A

## Abstract

**Purpose:**

To evaluate the ameliorative effect of mesenchymal stem cells (MSCs) on acetic acid colitis model via Nrf2/HO-1 pathway in rats.

**Methods:**

In this study, 30 rats were divided into three groups. Acute colitis was induced by rectal administration of 4% solution of acetic acid. MSCs were injected intraperitoneally in the treatment group.

**Results:**

Increased levels of tumor necrosis factor-α (TNF-α), pentraxin-3, and malondialdehyde (MDA) in colitis group were revealed biochemically. Increased level of TNF-α and decreased levels of Nrf2 and interleukin-10 (IL-10) were observed in rectum tissues. Increased fibrous tissue proliferation, vascularization and inflammatory cell infiltration were described in the colitis group. Significant improvement was observed in MSCs treated group histopathologically. Increased immunopositivity of TNF-α, vascular endothelial growth factor (VEGF) and CD68 markers was observed in the colitis group cells, and decreased level of this positivity was observed in MSCs treated group.

**Conclusions:**

Biochemical, histopathological and immunohistochemical results strongly support the ameliorative effect of MSCs against acetic induced colitis model via Nrf2/HO-1 pathway in rats.

## Introduction

An inflammation is one of the beneficial responses of the body against different types of infection. It plays an important role in the healing and repairing of infected tissues^1^. Inflammatory response is a protective mechanism of body, but the mediators released in the inflammatory process may lead to the damage of tissues. The continuous and incorrect response of inflammatory process may cause chronic form of inflammation that leads to autoimmune diseases like inflammatory bowel disease in patients^2^. Inflammatory mechanism reveals non-specific (innate) and specific responses (adaptive). Complex cell signaling pathways are related to these mechanisms, and many drugs have potential actions on these responses^3^.

Two types of macrophage mediators (M1 and M2) play important role in healing of tissues and resolving of autoimmune diseases. The initiation process of inflammatory response and mediation of tissue healing take place by M1^4^,^5^. M2 are important for the activation of anti-inflammatory cytokines including interleukin (IL)-10 and transforming growth factor-β (TGF-β),that play important role in the resolution of cells or tissues^5^-^7^. These macrophages mediate the inflammatory mediators (IL-1β, IL-12, IL-18, IL-23, and tumor necrosis factor-α – TNF-α) in the autoimmune diseases like inflammatory bowel disease^8^-^10^. Oxidative stress secondary to inflammation has a significant role in ulcerative colitis. The activation of inducible nitric oxide (iNOS) synthase and cyclooxygenase-2 (Cox-2) is responsible for the reactive oxygen species. Nuclear factor-kB is responsible to induce Cox-2 and also plays an important role in the pathogenesis of inflammatory diseases. Acetic acid induced colitis model has been reported in previous studies to evaluate the effect of protective agents^11^-^13^.

Etiology, histopathological findings, and inflammatory mediator pathways of acetic acid induced colitis are very much similar to the colitis in humans^14^. Chronic inflammation of intestine is the main cause of colitis^13^. The over-activation of lymphocytes and pro-inflammatory cytokines cause chronic inflammation by overcoming the effect of regulatory lymphocytes and anti-inflammatory factors including TGF-β and IL-10^15^.

The nuclear factor erythroid 2 (NFE2)-related factor 2 (Nrf2) is a transcription factor and has significant role in the inflammatory diseases. It plays a protective role against inflammatory diseases of different organs including stomach, colon, joints, lungs, heart, and brain. An important role of Nrf2 in the macrophages mediated defensive mechanism against lung inflammation has been reported^16^. Nrf2 regulates the expression of antioxidants against oxidative stress and inflammatory processes. The sequestration of Nrf2 by Kelch-like ECH-associated protein (Keap 1) takes place in cytosol part of cells. The mediation of ubiquitination and degradation processes of Nrf2 via CUL-E3 ligase takes place by Keap 1^17^. During oxidative stress and inflammatory processes, reactive oxygen species start to increase in cells, and then dissociation of Keap 1 takes place from CUL-E3 ligase. After that, Nrf2 translocation takes place into nucleus and it performs the process of transcription of target genes like heme oxygenase-1 (HO-1)^18^.

Mesenchymal stem cells (MSC) are multipotent cells and found in bone marrow and adipose tissues. These cells have ability to self-differentiate by the process of mitotic cell division^19^. Stem cells derived from the adipose tissues have higher rate of proliferation, then the bone marrow derived stem cells^20^. Stem cells migrate through endothelium and reside in damaged organs. MSC mediate immunomodulatory actions against the chemo-attractants of inflammatory process by the help of released chemokine receptors^21^. Soluble factors generated by MSC reveal positive role in the repairing and healing of tissues via differentiation process of MSC. This process takes place in different steps, and the name of this phenomenon is paracrine effect. Nitric oxide, IL-6, prostaglandin-E2, hepatocyte growth factor, 3 dioxygenase and heme oxygenase-1 and indoleamine 2 are the MSC generated soluble factors^22^. MSC have immunomodulatory^23^ and protective effect against oxidative stress and inflammation^24^.

The purpose of this study was to evaluate the ameliorative effect of MSC on acetic acid colitis model via Nrf2/HO-1 pathway in rats.

## Methods

Ethical approval was taken from the Science University with the number 26220300.

In this experimental protocol, there were three groups. Each group had 10 male Wistar albino rats with weight of 200-250 g. The rules of National Institutes of Health were followed for the use of animals. Rats were housed in temperature and humidity-controlled environment, and water and food were provided *ad libitum*. The temperature of the room was kept at 22±2 °C, and 12-h light/dark period was provided. Acetic acid was bought from Sigma company, and 4% solution was used for the induction of acute colitis. The volume of 1 mL for 20 rats was used. No chemical solution was given to control group.

The protocol of ether anesthesia was used for the rectal administration of acetic acid. Catheter was used for acetic acid administration, and it was taken out after the complete spread of acetic acid in rectum. It was taken care that no solution would be out from the anus. The normal saline solution (0.9% NaCl) was also given to the colitis group from the intraperitoneal route with the dose rate of 1 mL/kg. MSCs were given (2 × 10^6^ cell/kg) by the intraperitoneal route. This experiment was completed in 15 days. After the completion of experiment, dissection was performed with the help of general anesthesia (Ketasol 100 mg/kg) + xylazine 50 mg/kg) to collect the colon tissues and blood samples for histopathological and biochemical processes.

### Mesenchymal stem cells isolation from adipose tissue

MSCs isolation was done from the adipose tissues of flank region of rats with the help of same general anesthesia. Fat tissues were transferred to stem cell laboratory. Cold chain and sterile environment were maintained during this transfer. Tiny segments of these adipose tissues were treated with 0.2% collagenase type II (Gibco, United States of America). This procedure was done at room temperature for 40 minutes.

Centrifugation of lysed tissues was done at 1,500 rpm for 5 min. Culture flasks containing 3-mL modified eagles medium (DMEM; Gibco, United States of America) supplemented with 10% fetal bovine serum (FBS, Gibco, United States of America), 1% penicillin and 1% streptomycin (Sigma, United States of America), and 2-mM L-glutamine (Invitrogen, Netherlands) were used for the suspension of precipitate. These flasks were put in incubator having 5% CO_2_. Room temperature was maintained in incubator.

Three days later, this media was refreshed to take the confluence of 85%. 0.25% trypsin (Gibco, United States of America) was used for subculturing until the fourth passage, and later inactivation was done by adding DMEM. Cryopreservation of these isolated MSCs was done for the future use in transplantation processes by using × 10^6^ viable cells/mL in 50% DMEM media, 40% FBS, and 10% dimethyl sulfoxide (DMSO; MP Bio). Sterile cryovials were used for this purpose and kept in nitrogen cylinder with the temperature of -196 °C. Growth and morphology of cells were observed with the help of an inverted microscope. Thawing cryovials was done in water at room temperature. Centrifugation was done at 1,500 rpm for 5 minutes. Pellet of cells were suspended again in DMEM and safely placed in incubator at room temperature.

### Characterization of mesenchymal stem cells

For this purpose, immunofluorescence staining of CD13, CD29 and CD105 molecules was done for the characterization of MSCs during their second passage. Cells were grown in cultural dishes for immunostaining process. Phosphate buffer solution (PBS) was used for washing, and methanol was used for fixation purposes at the temperature of -10 °C. Methanol was removed after fixation, and desiccation was done. Normal goat serum was used for blocking of cells, and incubation was done for 20 minutes. Blocking serum was taken out, and cells were washed with the PBS solution. After that, isolation, and characterization of MSCs were done by simple technique. Incubation of cells was done after spreading primary antibodies of CD29, CD105 and CD13. Cells were washed again with PBS solution. Secondary antibody solution was spread on slides, and incubation of 45 minutes was applied. Washing of cells with PBS solution was done, and mounting medium was used for the clear visualization of cells under fluorescence microscope. Room temperature was maintained during all the processes.

### Histopathological evaluation of rectum

The size of 4 μm sections was taken from the paraffin blocks of tissues for the staining of hematoxylin and eosin. Axiocam digital camera was used to take photographs of sections. The criteria for the scoring were according to MacPherson and Pfeiffer’s criteria^25^:

0 = for normal epithelium, no hemorrhages or leukocytes infiltration;
1 = for less than 25% epithelium disruption and leukocytes infiltration;
2 = for the epithelium disruption of 25%, focal hemorrhages and leukocytes infiltration;
3 = for the 50% epithelium disruption, hemorrhages, and leukocytes infiltration;
4 = for more than 50% epithelium disruption and other pathological changes.

### Immunohistochemical evaluation of TNF, VEGF and CD68

The colon tissues were processed in routine processing method, and tissues were blocked in paraffin. Sections of 4 μm were taken on special adhesive slides for immunohistochemical evaluation. Slides were treated with 3% hydrogen peroxide solution for 20 minutes for quenching of endogenous peroxidases activities. Antigen retrieval was done with citrate buffer solution in a microwave oven at 90 °C for 15 minutes. Serum blocking solution Ultra V Block TA-060-UB (Thermo-Scientific) was applied on tissues for 20 minutes.

After that, primary antibodies of vascular endothelial growth factor (VEGF) (1/50 dilution, sc-7269, Santa Cruz Biotechnology), TNF-α (1/100 dilution, Santa Cruz Biotechnology sc-52746), and CD68 (1/100 dilution, Santa Cruz Biotechnology, SC-59103) were used and incubated for 24 hours at 4 °C. ABC kit (Vectastain Elite ABC-HRP Kit, PK-6101, Vector Laboratories) was used for detection of antibodies. 3-amino-9-ethylcarbazole (AEC, TA-125-HA, Thermo-Scientific) was used for the visualization process. All slides were photographed with an Axiocam ICc 5 digital camera, and images were analyzed with ZEN2 image analysis system and image J. Each case was evaluated separately by giving semi-quantitative numbers from 1 to 4 according to the intensity of staining:

0 = no staining;
1 = weak;
2 = moderate;
3 = strong.

Obtained data were analyzed by Statistical Package for the Social Sciences (SPSS) program.

### TNF-α level measurement in plasma

Enzyme-linked immunosorbent assay (ELISA) kit of Biosciences company was used for the detection of TNF-α levels in plasma. Dilution of plasma samples was done with dilution rate of 1:2. The guide of manufacturer company was used for the determination of TNF-α levels for two times. More than 2-pg/mL range was the significant detection range of TNF-α.

### Evaluation of plasma pentraxin-3 level

The ELISA apparatus at 450 nm by using pentraxin-3 (PTX-3) kit (Uscn Life Science Inc., Wuhan, China) determined the level of PTX3 in each 100-μL plasma sample. The guide of manufacturer company was used for the determination of PTX-3 levels for two times.

### Detection of TNF-α, Nrf-2, and IL-10 in rectum

One-mL of buffer solution having 1 mmol/L of PMSF, 1 mg/L of pepstatin A, 1 mg/L of aprotinin, and 1 mg/L of leupeptin in PBS solution (pH 7.2) was used in homogenizer for homogenization of rectal tissues. After that, centrifugation was done for 20 minutes at 12,000 rpm. Supernatant solution was used for further processes. Bradford method was used for the determination of total protein. ELISA kit was used for the determination of TNF-α, IL-10 and Nrf-2 levels.

### Lipid peroxidation measurement in plasma

Malondialdehyde (MDA) levels as thiobarbituric acid reactive substances (TBARS) were measured in plasma samples for the determination of lipid peroxidation. Trichloroacetic acid and TBARS reagent addition were done in plasma samples. After mixing these reagents, incubation was done for 1 hour at 100 °C. Ice was used for the cooling purpose. After that, centrifugation of samples was performed for 20 minutes at 3,000 rpm. Supernatant was used for the reading of absorbance at 535 nm. Tetraethoxypropane was used for calibration purpose.

### Statistical analysis

The SPSS version 15.0 (Chicago, IL, United States of America) was used to perform statistical analysis of this experimental study. Student’s t test was used to compare the group of parametric variables, and one-way analysis of variance (ANOVA) was used for analysis of variance. Mann-Whitney’s U test was used to compare the group of nonparametric variables. Data results were showed as mean ± standard error of the mean (SEM). P ≤ 0.05 value was considered as significant in this study.

## Results

### Biochemical results

The levels of TNF-α, PTX-3, and MDA were measured in plasma samples, and there was significant increase in colitis group. Significant improvement in the results were observed in the plasma level of inflammatory cytokines and MDA in MSCs treated group. TNF-α, Nrf2 and IL-10 concentration was measured in rectum tissues of all the groups. Control group revealed low level of TNF-α in both plasma and colon tissues. Significant increase was observed in the level of TNF-α in colitis group that was decreased after the treatment of MSCs. Nrf2 and IL-10 levels were also measured in rectum tissues, and the results were different. Control group revealed higher levels of both Nrf2 and IL-10. Downregulation was observed in the levels of both Nrf2 and IL-10 in the colitis group. After the treatment of MSCs, significant downregulation was observed in the levels of IL-10 and Nrf2 in the treatment group (Table 1).

**Table 1:** The results of biochemical parameters@.

Biochemical parameters	Control group	Colitis and % 0.9 NaCl (saline) given group	Colitis and MSCgiven group
Plasma TNF-α level (pg/mL)	16.7 ± 1.1	63.9 ± 2.7*	38.3 ± 5.4##
Plasma PTX-3 level (ng/mL)	0.74 ± 0.06	3.1 ± 0.15**	1.6 ± 0.09##
Plasma MDA level (nM)	58.5 ± 9.3	102.6 ± 12.6**	98.9 ± 7.5#
Rectum TNF-α level (pg/mg tissue)	61.1 ± 4.2	113.8 ± 9.2*	77.4 ± 6.6#
Rectum Nrf-2 level (pg/mg protein)	27.6 ± 3.5	10.3 ± 2.06**	19.2 ± 1.5#
Rectum IL-10 level (pg/mg protein)	1.08 ± 0.03	0.65 ± 0.02**	0.84 ± 0.02#

@ Data results were evaluated as mean ± standard error mean. One-way analysis of variance (ANOVA) was used;

* p < 0.05 significant difference from normal groups;

** p < 0.001 significant difference from normal groups;

# p < 0.01 significant difference from colitis and MSCs treated groups;

## p < 0.001 significant difference from colitis and MSCs treated groups; MSC: mesenchymal stem cells; TNF-α: tumor necrosis factor-α; PTX-3: pentraxin-3; MDA: malondialdehyde; IL: interleukine.

### Histopathological results

**Figure 1:**
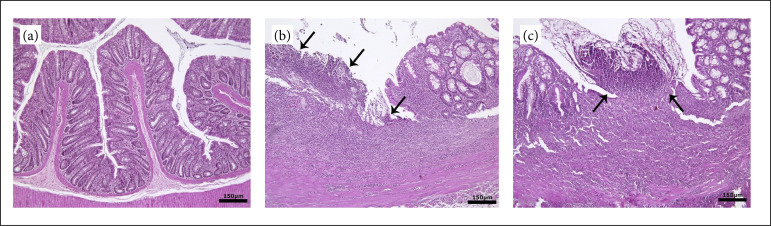
Microphotographs of colons exposed to acetic acid and treated with stem cells. **(a)** Control group,**(b)** severe ulcerative inflammation (*arrows*) in a large area of the colonic mucosa in group 1. **(c)** Mildulcerative inflammation (*arrows*) in group 2. Hematoxylin and eosin (H&E), bar = actual length.

No lesion was found in the control group. In group 1 (acetic acid), mucosal epithelium loss was observed in large areas in the samples. There was severe inflammatory cell infiltration consisting of neutrophil leukocytes, macrophages and lymphocytes descending from the lamina propria to the muscular layer. In these areas, fibrous tissue proliferation and vascularization were noted in places. In group 2 (acetic acid), similar lesions were found to be milder in the fields (Fig. 1). The lesions seen were scored according to Macpherson and Pfeiffer’s criteria^25^. The obtained data were evaluated statistically with the SPSS program (Table 2).

**Table 2:** The results of histopathological parameters*.

Groups	H&E results
Control group	0.00 ± 0.00
Colitis and % 0.9 NaCl (saline) given group	3.40 ± 0.69**
Colitis and MSC given group	2.10 ± 1.10#

* Data results were evaluated as mean ± standard error mean. One-way analysis of variance (ANOVA) was used;

** p < 0.001 significant difference from normal groups;

## p < 0.001 significant difference from colitis and MSCs treated groups; MSC: mesenchymal stem cells; H&E: hematoxylin and eosin.

### Immunohistochemical results

**Figure 2:**
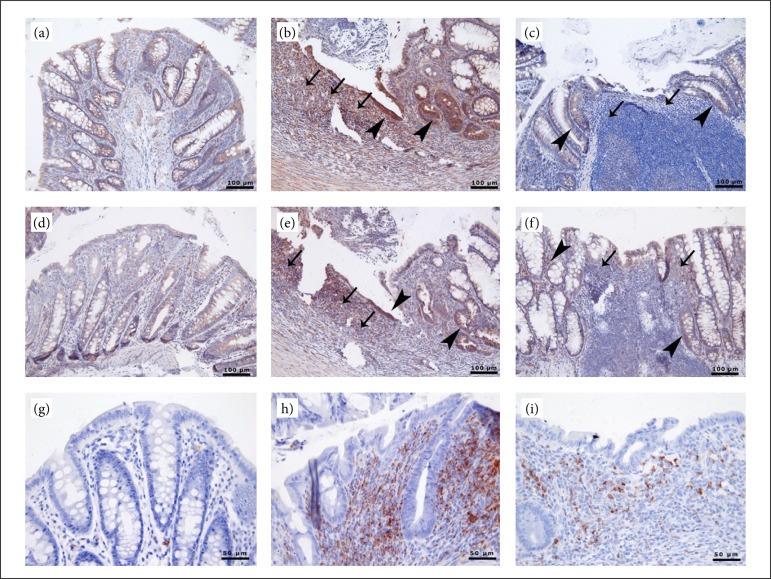
Immunohistochemical examination of the groups for VEGF, TNF and CD68. **(a-c)** VEGF: **(a)** Mild VEGF positivity in epithelia in the control group, **(b)** severe VEGF positivity in inflammatory cells (*arrows*) and epithelial cells (*arrowheads*) in group 1. **(c)** Decreased VEGF positivity in inflammatory cells (*arrows*) and epithelial cells (*arrowheads*) in group 2. **(d)** D-F: TNF-α: Mild TNF-α positivity in epithelia in the control group, **(e)** severe TNF-α positivity in inflammatory cells (*arrows*) and epithelial cells (*arrowheads*) in group 1. **(f)**Decreased TNF-α positivity in inflammatory cells (*arrows*) and epithelial cells (*arrowheads*) in group 2. **(g-i) (g)** CD68: few positive macrophages (*red cells*) in the control group, **(h)** many positive cells among inflammatory cells in group 1, **(i)** decreased CD68 positivity among inflammatory cells in group 2. ABC-peroxidase method, AEC chromogen, Gill’s hemalum. Bar = actual length.

VEGF: vascular endothelial growth factor; TNF: tumor necrosis factor.

In the examinations with VEGF and TNF, various levels of positivity were observed in intestinal epithelium, inflammatory cells in the region, vessels and muscular layers. In the examination performed with CD68, positive macrophages were noticed in both groups (Fig. 2). The results obtained from the groups are given in Table 3.

**Table 3:** The results of immunohistochemical evaluation*.

Groups	TNF	VEGF	CD68
Control group	0.70 ± 0.48	0.90 ± 0.32	0.00 ± 0.00
Colitis and % 0.9 NaCl (saline) given group	2.40 ± 0.70**	2.50 ± 0.71**	1.30 ± 0.67**
Colitis and MSC given group	1.10 ± 0.57##	1.50 ± 0.85##	0.70 ± 0.67##

TNF: tumor necrosis factor; VEGF: vascular endothelial growth factor; MSC: mesenchymal stem cells;

* data results were evaluated as mean ± standard error mean. One-way analysis of variance was used;

** p < 0.001 significant difference from normal groups;

^#^p < 0.01 significant difference from colitis and MSCs treated groups;

## p < 0.001 significant difference from colitis and MSCs treated groups.

## Discussion

Inflammation is a protective action against cell injuries resulting from the serious and original insults. The processes of cellular changes, infiltration of leukocytes to the injury site, and vascular changes like vasodilation contribute to inflammation. Ulceration may be found due to the response of chronic inflammation and necrosis of tissues^26^. The inflammation induced by acetic acid mediates the movement of acid into the epithelium cells and leads to the damage of epithelial cells^27^. Acetic acid experimental model for the induction of colitis is easy to conduct and has been reported in many previous studies^11^-^13^. Macroscopic changes in colon related to acetic acid include thickening of intestinal walls, ulceration, and necrosis. Mucosal lining damage, necrosis of tissues with hemorrhages, goblet cells loss, and infiltration of inflammatory cells have been reported in histopathological examination of colitis^12^,^13^,^28^. Biochemical results revealed significant increase in inflammatory cytokines IL-1β, TNF-α, IL-6 and also decrease in IL-10^29^. Histopathological and biochemical results of colitis model in our study resemble with the previous studies.

MSCs are the group of non-hematopoietic, self-renewing and fibroblast cells like the stromal ones^30^. They have ability to self-differentiate by the process of mitotic cell division^19^ into different cells.

Antioxidant, anti-inflammatory, and immunomodulating effects of MSCs have been reported in many studies^29^,^31^,^32^. In this study, the reformative effect of MSCs on acetic acid-induced colitis model via Nrf2/HO-1 pathway was evaluated by the help of histopathological, biochemical and immunohistochemical processes. Therapeutic effect of MSCs has been reported in which downregulation of immune and inflammatory actions (IL-2, TNF-α, IFN-γ, T-bet; IL-6, IL-17), and higher Th2 activities (IL-4, IL-10, GATA-3) were evaluated^33^. Significant improvement in the pathological changes in colitis model with the use of MSCs has been evaluated^33^,^34^. Protective and positive effects of MSCs have been reported in many previous studies of inflammatory model in colon. It was concluded that MSCs have antioxidant, anti-inflammatory and therapeutic effects^23^,^32^,^33^,^35^. The results of reformative effect of MSCs on the colitis model in rats of our study have similarities with the ones of previous studies.

The different pathways for the action of MSCs against colitis have been reported^13^,^28^, but we have evaluated the Nrf2/HO-1 pathway. Nrf2 is a transcription factor and it plays an important role in oxidative stresses^36^,^37^. It mediates the transcription of antioxidants and detoxification enzymes during oxidative stress in cells, and these enzymes play a critical role in the protection of cells against oxidative stress and cell injuries^38^-^40^.

Nrf-2 overexpression in MSCs decreases oxidative stress-induced apoptosis, and cytotoxicity has been reported^41^. Nrf2 plays an important role in the macrophages mediated defensive mechanism against lung inflammation^16^. The anti-inflammatory role of MSCs is related with changing the macrophages polarity^20^, and their anti-fibrotic effect may attribute to activate the Nrf2/HO-1 pathway for the defense mechanism against oxidative stress and inflammation. It can decrease the inflammatory processes through attenuation of NF-kB^42^,^43^. In our study, we suggested the reformative effect of MSCs via Nrf2/HO-1 pathway due to its anti-inflammatory and anti-fibrotic effects. Ameliorative effect of MSCs has also been reported against inflammation and fibrosis of liver through Nrf2/HO-1 pathway. This would be the first study to evaluate the reformative effect of MSCs on colitis model in which Nrf2 pathway was described. Higher level of Nrf2 was observed in the MSCs treated group when compared with the colitis group.

In this experimental study, higher level of TNF-α was found in the acetic acid treated colons of rats, and this level was decreased in the MSCs treated group. Similar to our results, TNF-α has been reported to play critical role in the pathogenesis of ulcerative colitis^44^,^45^. It can modulate the immune system, change the integrity of epithelium, and increase the infiltration of macrophages and neutrophils that are the main reason of colitis.

High level of immunopositive cells of TNF-α has been reported in acetic acid-induced colitis group^28^,^34^. Our results of TNF-α immunopositivity are similar with the previous studies. Other different markers have been used in immunohistochemical evaluation of colitis model including NF-kB^28^,^34^, PCNA and cox-2^34^,^46^,^47^, caspase-3^29^,^34^ and iNOS^34^,^47^. In this study, two different markers were also used that were not evaluated in previous studies of acetic acid induced-colitis model in rats.

The process of angiogenesis cause inflammation of vessels and support the disease activity and progression. Immune cells recruitment, inflammatory cells secretion and increased vascular permeability are the main activated components^48^,^49^. The increase in the density of vessels is related to the severity of disease in ulcerative colitis. VEGF is a main mediator for angiogenesis, and upregulation of VEGF in colitis has been reported in previous studies^50^-^52^. The immunopositive results of VEGF in our study have resemblance with the previous results. Macrophages infiltration into the colon mediated with the destruction of intestinal barrier that leads to colitis. CD68 is a marker for macrophages/monocytes in the colitis. The significant increase of CD68 expression has been reported in colitis^53^,^54^. Same results of higher immunoreactivity with CD68 were evaluated in the current study.

## Conclusion

All the histopathological, biochemical and immunohistochemical results of the current study strongly support the ameliorative effect of MSCs against colitis model via Nrf2/HO-1 pathway. It was concluded that MSCs has antioxidant, anti-inflammatory and immunomodulatory effect against acetic acid-induced colitis model in rats via Nrf2/HO-1 pathway.
